# Immunosenescence is associated with altered gene expression and epigenetic regulation in primary and secondary immune organs

**DOI:** 10.3389/fgene.2013.00211

**Published:** 2013-10-18

**Authors:** Corinne Sidler, Rafał Wóycicki, Yaroslav Ilnytskyy, Gerlinde Metz, Igor Kovalchuk, Olga Kovalchuk

**Affiliations:** ^1^Department of Biological Sciences, University of LethbridgeLethbridge, AB, Canada; ^2^Department of Neuroscience, Canadian Center for Behavioural Neuroscience, University of LethbridgeLethbridge, AB, Canada

**Keywords:** immunosenescence, gene expression profile, histone modification, DNA methylation, genome instability, aging

## Abstract

Deterioration of the immune system (immunosenescence) with age is associated with an increased susceptibility to infection, autoimmune disease and cancer, and reduced responsiveness to vaccination. Immunosenescence entails a reduced supply of naïve T cells from the thymus and increased specialization of peripheral T cell clones. Both thymic involution and peripheral T cell homeostasis are thought to involve cellular senescence. In order to analyze this at the molecular level, we studied gene expression profiles, epigenetic status, and genome stability in the thymus and spleen of 1-, 4-, and 18-month-old Long Evans rats. In the thymus, altered gene expression, DNA and histone H3K9 hypomethylation, increased genome instability, and apoptosis were observed in 18-month-old animals compared to 1- and 4-month-old animals. In the spleen, alterations in gene expression and epigenetic regulation occurred already by the age of 4 months compared to 1 month and persisted in 18-month-old compared to 1-month-old rats. In both organs, these changes were accompanied by the altered composition of resident T cell populations. Our study suggests that both senescence and apoptosis may be involved in altered organ function.

## Introduction

The adaptive immune system provides protection against a wide variety of pathogens and cancer. A fine balance between positive selection (T cell activation by antigen) and negative selection (limitation of autoreactive T cells) is in place to avoid autoimmune reactions. However, these selection processes are energy-demanding and inefficient—more than 95% of the initially produced T cells are subsequently eliminated (Surh and Sprent, 1994). Therefore, after peripheral lymphatic organs have been populated with T cells, the thymus starts decreasing in size, weight, and cellularity initially by about 3% per year and after the 5th decade of life by about 1% per year in humans (Steinmann et al., 1985). This shrinkage of the thymus, also termed the age-associated thymic involution, is evolutionarily conserved in species with a thymus. It is accompanied by a change in thymus architecture resulting in the reduced thymic output of naïve T cells proportional to thymus size (Cunningham et al., 2001).

The decline in thymic T cell export along with a reduced proliferation of peripheral naïve T cells and altered composition of T cell populations contribute to the establishment of immunosenescence, which is linked with higher morbidity and mortality in the elderly (Ferguson et al., 1995).

The driving force of age-associated involution is thought to lie within the epithelial compartment of the thymus itself (Ortman et al., 2002; Zhu et al., 2007; Aw et al., 2009). Further, there is some evidence that apoptotic and senescent cells accumulate in the epithelial compartment during thymic involution (Aw et al., 2008), which results in a shrinkage of the epithelial compartment (Flores et al., 1999). Thus, thymic involution may be driven by similar molecular mechanisms as the aging process in other organs.

Despite decreasing thymic output, the T cell pool in the periphery is relatively constant in size, which can be explained by the expansion of peripheral T cell populations (Mackall et al., 1993). Mackall et al. (1993) showed that in the presence of a functional thymus, the peripheral expansion of T cells was suppressed. The peripheral expansion is driven by interactions with antigen and therefore leads to an increasing adaptation to the individual's environment and decreasing responses to new antigens (Mackall et al., 1996). After several rounds of clonal selection, T cells can become replicatively senescent *in vitro*, and T cells with senescence-like features are also found *in vivo* (Effros, 2004).

However, the molecular mechanisms that underlie those changes are only beginning to be understood. Altered expression and activity of several transcription factors are involved in thymic involution (Trebilcock and Ponnappan, 1996; Ortman et al., 2002). This suggests that transcriptional profiles in tissues of the aging immune system may be altered.

Further, senescence plays a role in thymic involution as well as in homeostasis of peripheral T cells. At a molecular level, cellular senescence is often linked with the accumulation of oxidative damage to macromolecules (including DNA as the genetic material and chromatin as the substrate for epigenetic regulation). While the accumulation of mutations has long been hypothesized to be a cause of aging, damage to chromatin has recently been suggested to be involved in aging as well (Sedivy et al., 2008).

Therefore, we hypothesized that if senescence plays a role in immunosenescence, gene expression and epigenetic profiles may be vastly altered in primary and peripheral immune organs of aging organisms. To assess this, we isolated thymus and spleen tissues from 1-month, 4-month (before or at an early stage of thymic involution) and 18-month-old (at a late stage of thymic involution) male Long Evans rats. Using the Illumina® Gene Expression BeadChip technology, we determined transcript levels in total RNA preparations from both organs. Here, we report that along with profoundly altered gene expression profiles, both in the thymus and spleen, transcriptional and epigenetic regulation are affected with increasing age. This is accompanied by altered expression of CD surface markers and the composition of T cell populations in both organs.

## Results

### Age-dependent gene expression changes do not occur simultaneously in different organs

**Figure 1:**
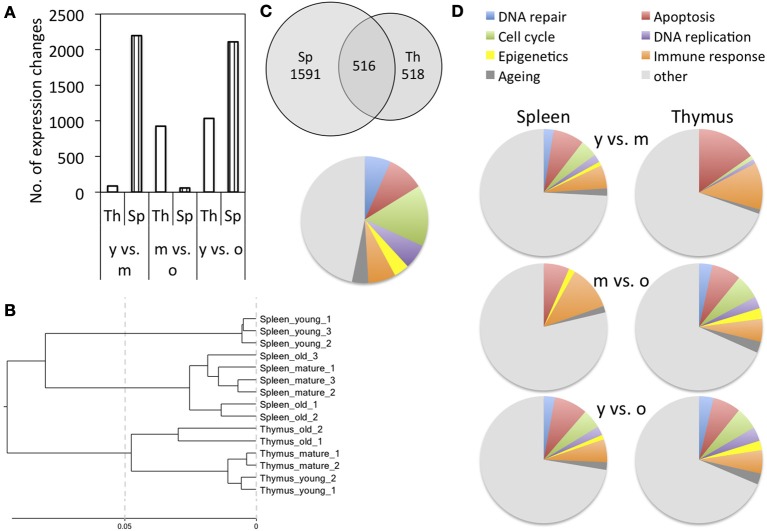
**Cluster analysis and functional classification of gene expression results**. **(A)** Number of differentially expressed genes when comparing different age groups; y is young, m is mature, and o is old. **(B)** Cluster analysis based on all probes represented on the BeadChip. **(C)** Overlap of gene expression changes observed in the spleen and thymus of young and old animals and functional classification of the genes that were commonly differentially expressed. **(D)** Functional classification of genes affected by differential expression.

To get an understanding of age-dependent changes that occur in primary and secondary immune organs, we profiled mRNA transcripts from thymus and spleen tissues extracted from 1-month (young), 4-month (mature) or 18-month-old male Long Evans rats using Illumina® RatRef12 BeadChips (S1). The number of genes affected by expression changes varied with age and tissue. In thymus, changes in the expression of 1034 genes were detected between young and old animals, whereas only 86 genes were affected between 1-month and 4-month-old animals. In spleen, high numbers of expression changes were observed when comparing old and mature animals to young animals (2196 and 2019 genes, respectively), whereas a low number of changes occurred between 4- and 18-month-old animals (Figure 1A). The cluster analysis based on all probes represented on the BeadChip further showed that for spleen, expression profiles of mature and old animals clustered more closely, whereas for thymus, profiles of young and mature animals clustered more closely (Figure 1B).

When comparing the total gene expression changes that occur between young and old animals in spleen and thymus, 516 genes are differentially expressed in both tissues, whereas 1591 are spleen-specific and 518 thymus-specific changes (Figure 1C). The genes that were commonly differentially expressed in both spleen and thymus affected biological processes of cell cycle, DNA replication, immune response and epigenetics (Figures 1C, S2).

For better understanding of the functional implications of these expression changes, functional classification was performed. In both tissues, aging was associated with an increase in the number of differentially expressed genes involved in cell cycle regulation, DNA replication, and senescence. Alongside those, genes involved in DNA repair, epigenetic regulation, apoptosis, and immune response were also increasingly affected by age-dependent differential expression (Figures 1D, S2).

### Changes in cell cycle regulation and senescence gene expression indicate that older tissues contain more senescent cells

Functional classification showed increasing fractions of differentially expressed senescence-related genes in mature and old tissues in spleen, and old tissues in thymus, as compared to young tissues (Figure 1D). This may be explained by an accumulation of senescent cells in both tissues with age, which is in line with previous observations that senescent cells accumulate in the thymus of aging animals (Aw et al., 2008) and among peripheral T cells (Effros, 2004).

Key features of senescent cells include cell cycle arrest in G0/G1 phase (Sherwood et al., 1988) as well as distinct changes to gene expression profiles (de Magalhaes et al., 2009). In order to study cell cycle status of thymus and spleen samples, genes involved in cell cycle regulation were further subcategorized into regulators of cell cycle progression, genes involved in the progression of M, G1, S, and G2 phases of the cell cycle (Table 1). As a general trend, it was observed that a high number of genes involved in cell cycle regulation are down-regulated irrespective of whether the function of those genes is either to promote or inhibit cell cycle progression.

**Table 1:** **Expression changes of cell cycle regulators**.

**Sub-Category**	**Thymus**	**Spleen**
Cell cycle progression		
Inhibitors ↑	0	14
Activators ↓	80	67
G1 progression		
Inhibitors ↑	25	36
Activators ↓	86	37
S phase progression ↓	100	93
DNA replication ↓	97	93
G2 progression ↓	75	50
Mitosis ↓	91	89

Percentages of up- or down-regulated genes compared to the total number of genes with expression changes in the according subcategory. Arrows indicate whether the represented numbers reflect the fraction of up- or down-regulated genes.

Among them, several cyclin genes (*Ccna2, Ccnb2*, and *Ccne2*) were down-regulated in the spleen of mature and old animals and in the thymus of old animals, whereas *Ccnd1* was up-regulated in those tissues. Furthermore, *Cdc2*, which is important for G2/M transition and M progression, was down-regulated in the spleen of mature and old animals and in the thymus of old animals. In addition to those changes, in the spleen, *Cdk2* and *Cdk4* were down-regulated with increasing age. This indicates that in the spleen cells of mature and old animals and in the thymus cells of old animals, the potential for active cell cycling is limited.

**Figure 2:**
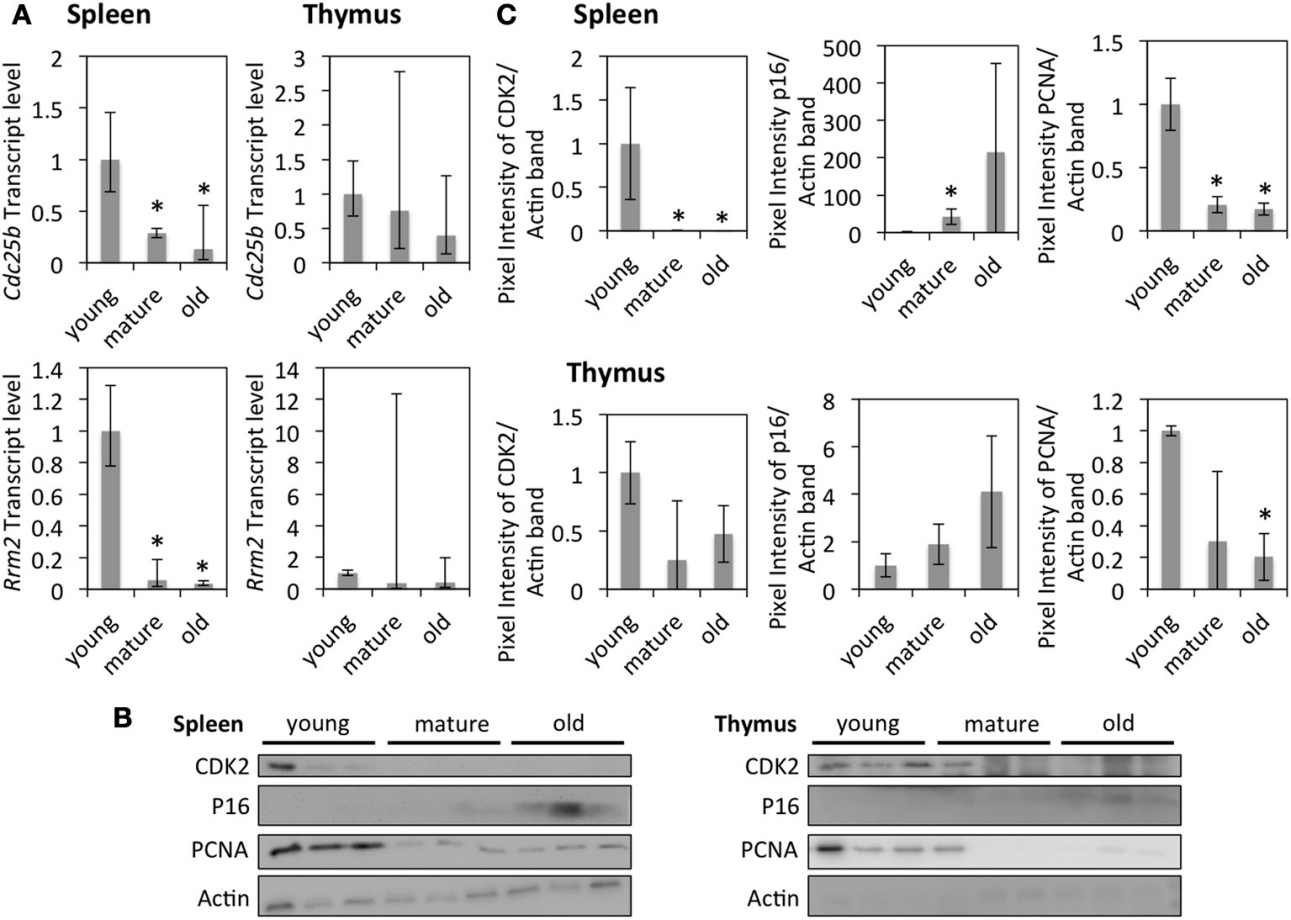
**The effects of age on cell cycle and DNA replication in the spleen and thymus of rats**. **(A)** Fold changes of mRNA expression as measured by qRT-PCR standardized to gene expression in young animals. The bars represent mean ± 95% confidence interval. **(B)** Western blots showing protein levels of CDK2, p16, and PCNA. **(C)** Protein expression levels of selected genes standardized to the expression in young animals and normalized to the expression of Actin. The error bars represent standard deviations. ^*^Indicates significance based on Student's t-test (*p* < 0.05) in panel **(C)**. ^*^Indicates significance based on one-way ANOVA (*p* < 0.05) in panel **(A)**.

Expression levels of several genes were confirmed at the RNA level by qRT-PCR or at the protein level by Western blot (Figures 2A–C). The down-regulation of *Rrm2* at the RNA level and PCNA at the protein level supports that DNA replication occurs in a smaller fraction of cells in older tissues. The down-regulation of *Cdk2* and up-regulation of p16^INK4a^ suggest that G1 progression occurs in a smaller fraction of cells in older tissues.

**Figure 3:**
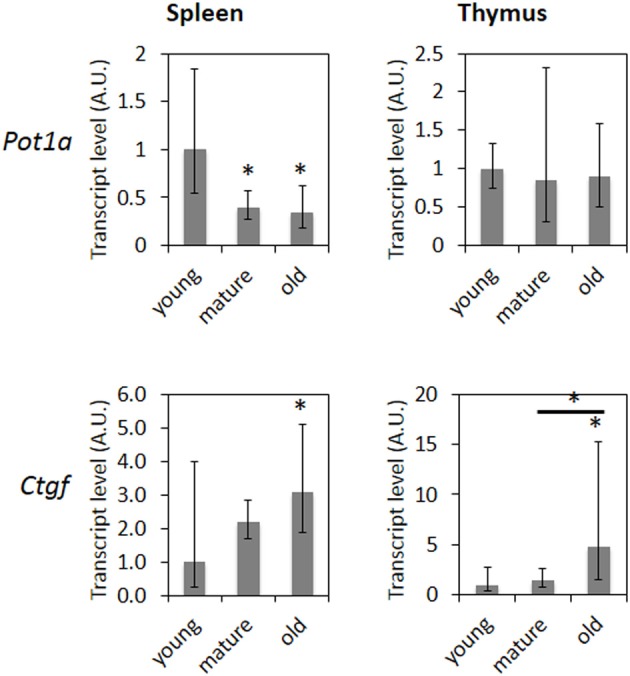
**mRNA levels of senescence-related genes in aging spleen and thymus**. Bars represent averages from three animals and two technical repeats normalized to expression in young animals ± 95% confidence intervals. ^*^Indicates significance based on one-way ANOVA (*p* < 0.05).

In respect to age-related gene expression changes, 18 out of 25 genes in the thymus and 35 out of 45 genes in the spleen showed expression changes consistent with previously reported age-related changes (de Magalhaes et al., 2009) (Table 2). Transcript levels of *Ctgf* and *Pot1a* were determined using qRT-PCR. *Ctgf* showed a significantly increased expression in the spleen of old and mature animals and in the thymus of old animals, whereas *Pot1a* transcript levels were significantly decreased in the spleen of mature and old animals (Figure 3).

**Table 2:** **Senescence-associated gene expression**.

		**Thymus**			**Spleen**		
**Gene**	**Involvement in aging**	**y-m**	**y-o**	**m-o**	**y-m**	**y-o**	**m-o**
Ada	Modulates Tert activity	N.S.	−0.7	−0.6	0.6	0.9	N.S.
Adipoq	May prevent senescence	N.S.	N.S.	3.3	N.S.	N.S.	N.S.
Anxa3	Up-regulated with age	N.S.	1.1	1.0	1.1	1.1	N.S.
Brd7	Mediates p53-induced senescence	N.S.	−0.7	−0.6	−0.6	−0.7	N.S.
C1qa	Up-regulated with age	N.S.	1.5	1.0	1.2	1.3	N.S.
C1qb	Up-regulated with age	N.S.	1.6	1.3	1.0	0.9	N.S.
C1qc	Up-regulated with age	0.6	2.0	1.4	1.3	1.3	N.S.
Cbx7	Represses Cdkn2a, extends lifespan	N.S.	0.6	N.S.	0.8	0.8	N.S.
Ccl5	Secreted by senescent fibroblasts	N.S.	N.S.	N.S.	1.4	1.0	N.S.
Cdkn1b	=p27Kip1; up-regulated with age	N.S.	−0.6	N.S.	N.S.	−0.6	N.S.
Clu	Up-regulated with age	N.S.	2.7	2.8	0.8	0.8	N.S.
Col1a1	Down-regulated with age	N.S.	N.S.	N.S.	N.S.	−0.9	N.S.
Col3a1	Down-regulated with age	−1.3	N.S.	N.S.	N.S.	−0.9	N.S.
Ctgf	Up-regulated with age	N.S.	1.8	1.2	1.3	1.9	N.S.
Ctss	Up-regulated with age	N.S.	1.4	1.0	1.5	1.7	N.S.
Cx3cl1	Down-regulated with age	N.S.	N.S.	N.S.	N.S.	0.6	N.S.
Eif5a	Up-regulated with senescence	N.S.	−0.6	N.S.	N.S.	N.S.	N.S.
Ets1	Regulates genes involved in senescence	N.S.	−1.0	−0.9	1.3	0.9	N.S.
Fcgr2b	Up-regulated with age	N.S.	0.9	N.S.	1.3	1.3	N.S.
Foxo1a	Involved in G1/G0 transition	N.S.	N.S.	N.S.	0.7	N.S.	N.S.
Gbp2	Up-regulated with age	N.S.	0.8	0.7	1.9	1.8	N.S.
Gfap	Up-regulated with age	N.S.	N.S.	N.S.	−2.0	−2.2	N.S.
Gns	Up-regulated with age	N.S.	0.9	0.9	1.0	1.3	N.S.
Hcst	Up-regulated with age	N.S.	N.S.	N.S.	1.1	1.2	N.S.
Id2	Down-regulated with senescence	N.S.	N.S.	N.S.	0.9	1.0	N.S.
Igj	Up-regulated with age	2.6	N.S.	N.S.	1.7	2.5	N.S.
Il15	Involved in ageing of immune system	N.S.	N.S.	N.S.	0.9	0.9	N.S.
Il1b	Changed expression in senescent cells	N.S.	N.S.	N.S.	1.5	1.6	N.S.
Laptm5	Up-regulated with age	N.S.	N.S.	N.S.	1.0	1.0	N.S.
Lgals3	Up-regulated with age	N.S.	N.S.	N.S.	0.6	1.0	N.S.
Litaf	Up-regulated with age	N.S.	N.S.	N.S.	0.8	0.6	N.S.
LOC500054	=Pot1a; involved in telomere maintenance	N.S.	−0.6	−0.7	N.S.	−0.7	N.S.
Lrp1	Senescence-related gene expression changes	N.S.	N.S.	N.S.	N.S.	0.7	N.S.
Map2k1	Is required for p53-induced senescence	N.S.	N.S.	N.S.	0.6	0.9	N.S.
Mgst1	Up-regulated with age	N.S.	2.6	2.0	1.1	0.9	N.S.
Mmp9	Regulates TERT and p16 expression	N.S.	N.S.	−0.7	N.S.	N.S.	N.S.
Mpeg1	Up-regulated with age	N.S.	N.S.	N.S.	1.1	1.2	N.S.
Msn	Up-regulated with age	N.S.	N.S.	N.S.	0.9	0.8	N.S.
Nbn	Telomere maintenance	N.S.	N.S.	N.S.	−0.7	N.S.	N.S.
Ndrg1	Up-regulated with age	N.S.	0.6	N.S.	0.7	N.S.	N.S.
Nfkb2	Age-related gene expression changes	N.S.	N.S.	N.S.	1.0	1.0	N.S.
Npc2	Up-regulated with age	N.S.	0.6	N.S.	0.9	0.7	N.S.
Pcsk6	Up-regulated with age	N.S.	N.S.	N.S.	−0.6	N.S.	N.S.
Psmd11	Up-regulated with age	N.S.	N.S.	N.S.	−0.6	−0.7	N.S.
Rad54l	Telomere maintenance	N.S.	−0.8	−0.6	−1.3	−1.4	N.S.
RGD1305645	Induces senescence	N.S.	N.S.	N.S.	2.0	2.1	N.S.
S100A4	Up-regulated with age	N.S.	N.S.	N.S.	N.S.	0.6	0.6
Serping1	Up-regulated with age	N.S.	N.S.	N.S.	1.0	1.2	N.S.
Sirt2	Natural ageing	N.S.	N.S.	N.S.	−0.6	−0.9	N.S.
Suv39h1	H3K9methylation dependent induction of senescence	N.S.	−0.9	−0.7	−1.2	−1.2	N.S.
Terc	Telomere maintenance	N.S.	N.S.	−0.6	−1.8	N.S.	N.S.
Tfrc	Down-regulated with age	N.S.	N.S.	N.S.	−2.2	−2.8	N.S.
Txnip	Up-regulated with age	N.S.	0.6	0.6	0.8	0.6	N.S.
Vwf	Up-regulated with age	N.S.	N.S.	N.S.	−1.4	−1.6	N.S.

Entries up-/down-regulated with age are based on microarray signatures from Human Ageing Genomic Resources (de Magalhaes et al., 2009). Other gene information is based on Genecards (www.genecards.org/; Safran et al., 2010). Values represent log2-fold changes in detected transcript levels; N.S. indicates a non-significant difference in transcript levels.

Fridman and Tainsky (2008) summarized the pathways affected by gene expression changes observed in several senescence and immortalization studies and reported that some of the key regulatory genes were involved in several pathways: pRB/p53, cytoskeletal, interferon-related, insulin growth factor related, MAPK and oxidative stress pathways (Fridman and Tainsky, 2008). Our results are consistent with their findings: 16 genes involved in p53 signaling and 8 genes involved in MAPK signaling are differentially expressed in the spleen between young and old animals. Like Ning et al. in their study (Ning et al., 2003), in our data, we did not find an overrepresentation of genes that were located in proximity to telomeres, indicating that there was no location effect of telomeres on gene expression in this model system.

In summary, age-related gene expression changes are observed in the spleen of mature and old animals and in the thymus of old animals. Further, gene expression changes affecting cell cycle progression are also observed in the same tissues, suggesting that higher ratios of cells are arrested in G1/G0 phase of the cell cycle. These findings support the assumption that there is a higher ratio of senescent cells present in the spleen of mature and old animals and in the thymus of old animals.

### A potential role of transcription factors in regulating age-dependent gene expression profiles

The fact that high numbers of genes, including many transcription factors, were differentially expressed with increasing age in both tissues indicates a role of transcription factors in shaping the gene expression profiles observed. In order to address this, promoter regions of differentially expressed genes were screened for the occurrence of transcription factor binding sites (TFBS) of transcription factors with altered transcript levels.

Some transcription factors were differentially expressed in both tissues (Table 3), however, in opposite directions. The 4 transcription factors affected by gene expression changes in both organs—*Bcl6, Myc, Tcf7, Ets1*—were down-regulated in the thymus but up-regulated in the spleen. In addition, there were several transcription factors that were tissue-specific.

**Table 3:**
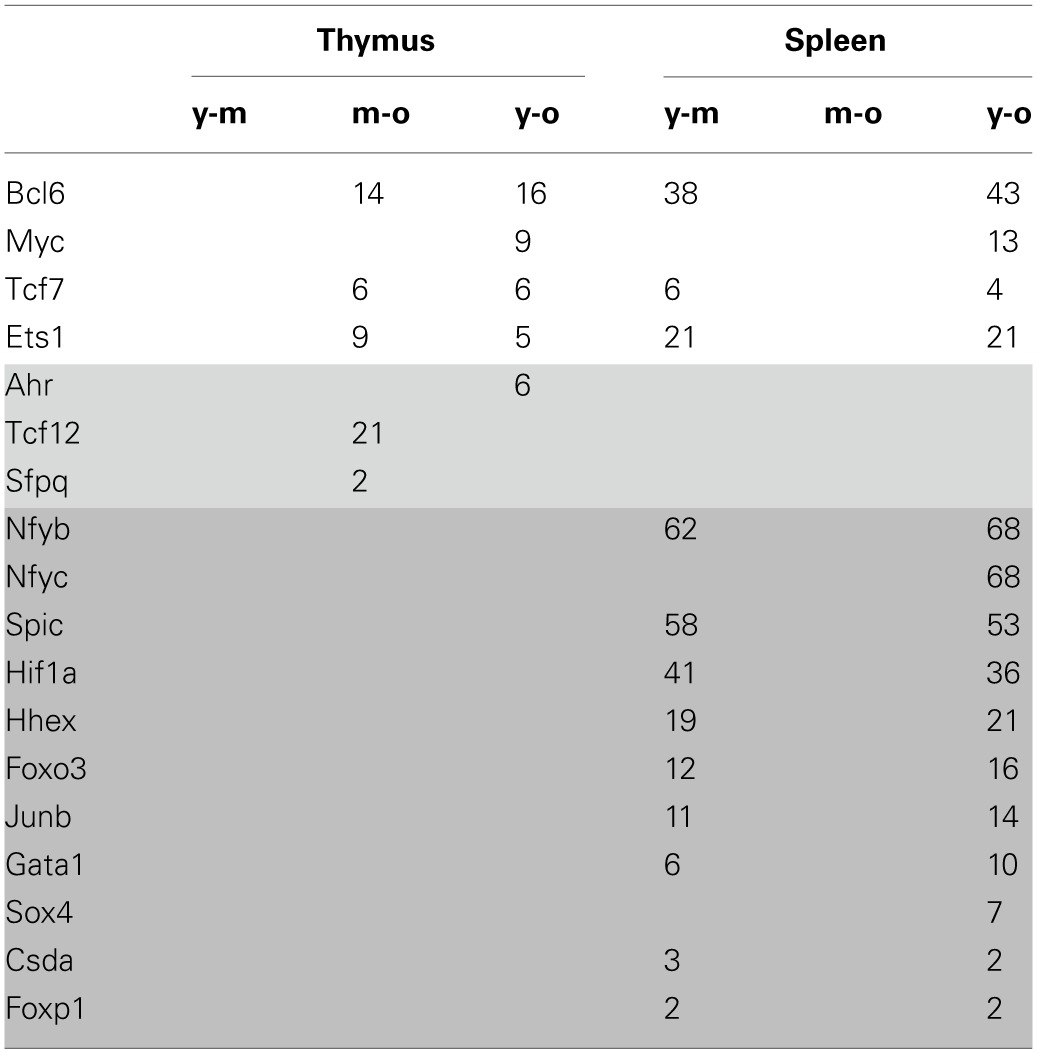
**The correlation of transcription factor expression with gene expression pattern**.

Number of target genes that correlate with transcription factor expression. The transcription factors shaded in white are commonly de-regulated in the thymus and spleen, light gray shading indicates the transcription factors with a thymus- specific expression pattern, and dark gray shading indicates transcription factors with a spleen-specific expression pattern.

Genes that had specific TFBS and were regulated in a similar manner to these transcription factors (either up- or down-regulated) were further classified according to their function. The predicted targets of the BCL6 transcription factor in the spleen were involved in cell cycle regulation and DNA replication; the predicted targets of SPIC and GATA1 were involved in lysosomal gene expression; and the predicted targets of HIF1α in chemokine signaling and cancer and insulin signaling pathways. For all other transcription factors considered, no overrepresentation of KEGG pathways among the target genes was detected by the DAVID software. Precise roles of diverse transcription factors in senescence and immunosenescence need further study.

### Regulation of DNA methylation and histone modification is altered in an age-dependent manner

Another factor, which is known to affect gene expression pattern is epigenetic regulation. The analysis of gene expression data revealed age-dependent down-regulation of DNA methyltransferases, histone modifying enzymes, chromatin remodeling enzymes, and miRNA/siRNA processing factors, suggesting that epigenetic regulation in old tissues is altered compared to that in young tissues.

**Figure 4:**
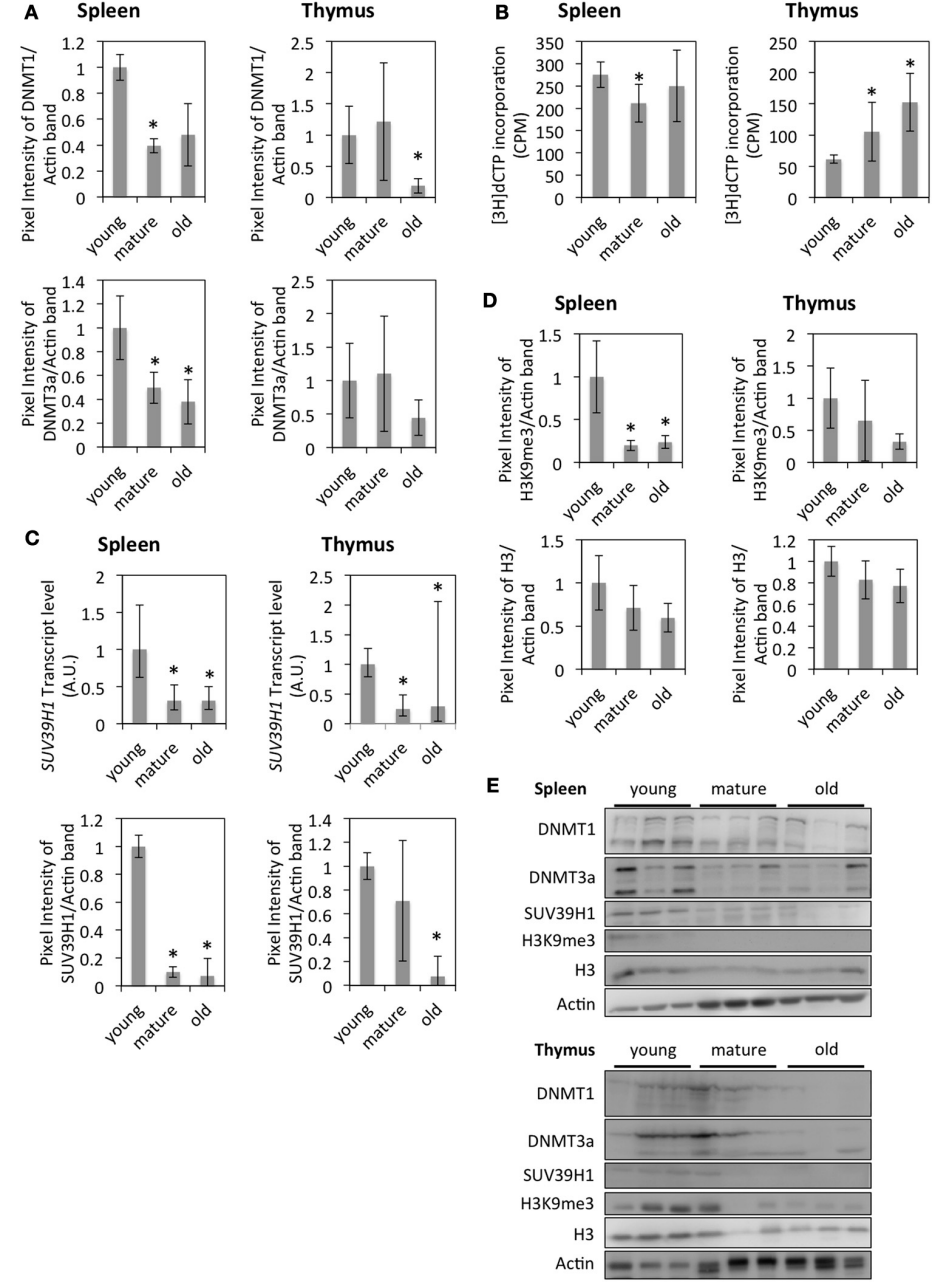
**Changes in epigenetic regulation in the thymus and spleen of aging rats**. **(A)** Protein levels of DNMT1 and DNMT3a. The bars represent means normalized to Actin expression and expression in young animals ± standard deviation. **(B)** Global DNA methylation levels as measured by the Cytosine Extension assay. The bars represent mean ± standard deviations. **(C)** RNA and protein expression levels of histone modifying enzymes as measured by qRT-PCR or Western blot. For qRT-PCR graphs, the bars represent mean standardized to expression levels in young animals. The error bars represent 95% confidence intervals. For Western blots, the bars represent means normalized to Actin bands and expression in young animals ± standard deviation. **(D)** Quantification of histones and histone modifications by Western blot. The bars represent means normalized to Actin bands or Coomassie loading controls and expression levels in young animals ± standard deviations. **(E)** Western blot images. ^*^Indicates significance based on Student's t-test (*p* < 0.05) in panels **(A,B,D)**. ^*^Indicates significance based on one-way ANOVA (*p* < 0.05) in panel **(C)**.

Down-regulation of DNMT1 and DNMT3a in the thymus and spleen of older animals was confirmed by Western blot analysis (Figures 4A,E); lower levels of DNMT1 and DNMT3a correlated with genome hypomethylation in the thymus of older animals as determined by a cytosine extension assay (Figure 4B). The difference in DNA methylation levels between spleens of young and old animals was not significant, whereas spleens of mature animals exhibited slight, but significant global DNA hypermethylation compared to tissues from young animals (Figure 4B).

It has been suggested elsewhere that with the onset of senescence, regions of constitutive heterochromatin become de-heterochromatinized, while senescence-associated heterochromatin foci are formed in regions of facultative heterochromatin (Zhang and Adams, 2007). Therefore, we hypothesized that global DNA hypomethylation in the thymus of old animals may be associated with the reactivation of transposable elements. Western blot analysis showed slightly increased expression levels of LINE1 ORF1 in the spleen and thymus of old animals; however, no increase in transcript levels could be detected (data not shown).

In addition to DNA methylation, post-translational modifications of core histone proteins play an important role in epigenetic control of gene expression. *Suv39h1*—a histone methyltransferase—was found to be down-regulated in the spleen and thymus of old animals, which was confirmed at the RNA level by qRT-PCR and at the protein level by Western blot (Figures 4C,E). Lower expression levels of *Suv39h1* also correlated with reduced global levels of trimethylated Histone 3 (H3K9me3) which was significant in spleen samples (Figures 4D,E). SIRT2—a histone deacetylase—showed lower expression levels in the spleen of older animals, which did not correlate with acetylation levels of H4K16—a SIRT2 target S3).

Both global DNA hypomethylation and histone hypomethylation indicate an increase in open chromatin and potentially genome instability, particularly in the thymus of mature and old animals.

### Tissues in older animals exhibit decreased expression of DNA repair genes and DNA damage signaling genes

**Figure 5:**
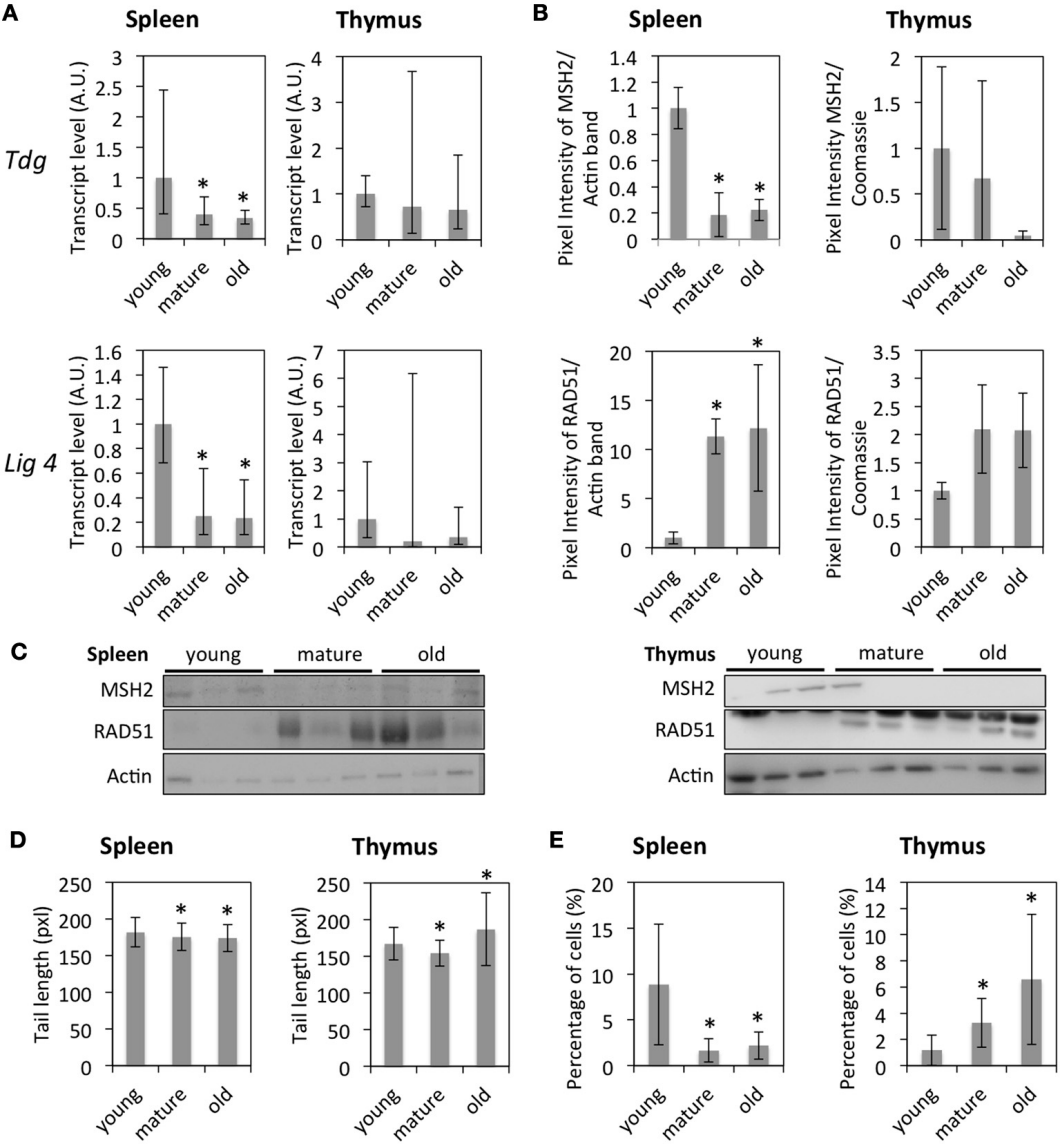
**Age-dependent changes in DNA damage and repair. (A)** mRNA fold expression changes normalized to a set of housekeeping genes and the expression in young animals as determined by qRT-PCR. The bars represent means ± 95% confidence intervals. **(B)** Protein expression levels for selected genes measured by Western blot. The bars represent means normalized to the Actin expression levels and the expression in young animals ± standard deviation. **(C)** Western blot images. **(D)** Olive comet tail lengths determined by the comet assay. The data points represent averages of 300–400 comets per age, the error bars show standard deviation. **(E)** Percentage of cells positive for γH2AX. The bars represent averages of 300–400 cells per age and organ ± standard deviations. ^*^Indicates significance based on one-way ANOVA (*p* < 0.05) in panels **(A,B,D,E)**.

Since global DNA hypomethylation and histone hypomethylation were observed in the thymus of old animals and histone hypomethylation—in the spleen of old animals, we hypothesized that tissues from old animals would exhibit an increased genomic instability. Gene expression data further indicated the down-regulation of genes involved in DNA damage signaling (*H2AX, Usp3, Bard1*, and others), response to oxidative stress (*Gpx1, Gpx4, Prdx2, Prdx3, Sod1, Cat* in spleen only), base excision repair (*Fen1, Lig1*), nucleotide excision repair (*Rpa2, Rpa3, Rad23a*), double-strand break repair (*Lig4, Rad54l, Trip13, Rad21*) and mismatch repair (*Tdg, Exo1, Msh2*, and *Msh6*). Expression levels of *Tdg* (MMR) and *Lig4* (NHEJ) were confirmed by qRT-PCR (Figure 5A) at the RNA level, and expression levels of MSH2 (MMR) and RAD51 (HR) by Western blot (Figures 5B,C) at the protein level. However, in the case of RAD51, while reduced transcript levels were detected in the spleen of old animals using the BeadChip technology, protein levels were found to increase with age.

The overall down-regulation of DNA repair enzymes at the transcript and protein levels suggested an increasing amount of unrepaired DNA damage in tissues of older animals. To study this, cells were isolated from the thymus and spleen of animals of different age groups and subjected to the Comet assay. The results indicated a slightly, but significantly increased level of unrepaired DNA damage in the thymus of old animals compared to that of young animals, but a slightly, but significantly decreased level of unrepaired DNA damage in the spleen of mature and old animals as well as in the thymus of mature animals (Figure 5D).

**Figure 6:**
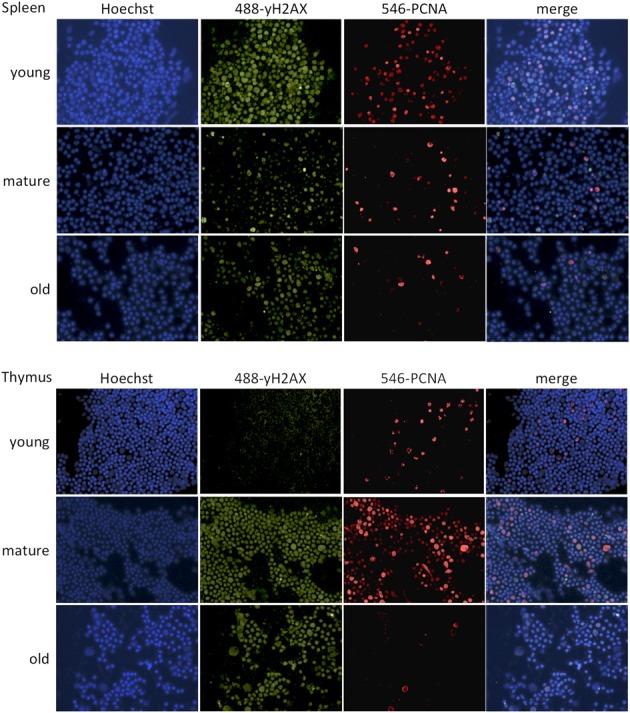
**Representative images of γH2AX-PCNA immunofluorescence staining**. Images were taken at 40x magnification.

In order to study changes in DNA damage signaling, the occurrence of γH2AX foci was determined by immunofluorescence staining (Figures 5E, 6). In line with the results of the Comet assay, the percentage of cells with γH2AX foci increased with age in the thymus and decreased in the spleen, thus correlating with global genome hypomethylation in the thymus of old animals.

### The thymus of older animals contains more apoptotic cells than in younger animals, but no clear trend is observed in the spleen

Changes in the expression of genes involved in apoptosis were different in thymus and spleen tissues, including both the induction and repression of both pro- and anti-apoptotic genes. In the thymus, 59% of pro-apoptotic genes were up-regulated, and 54% of anti-apoptotic genes were down-regulated, whereas in the spleen, 66% of pro-apoptotic genes were up- regulated and 50% of anti-apoptotic genes were down-regulated. Based on this, a slightly increased incidence of apoptotic cells in older tissues might be expected.

**Figure 7:**
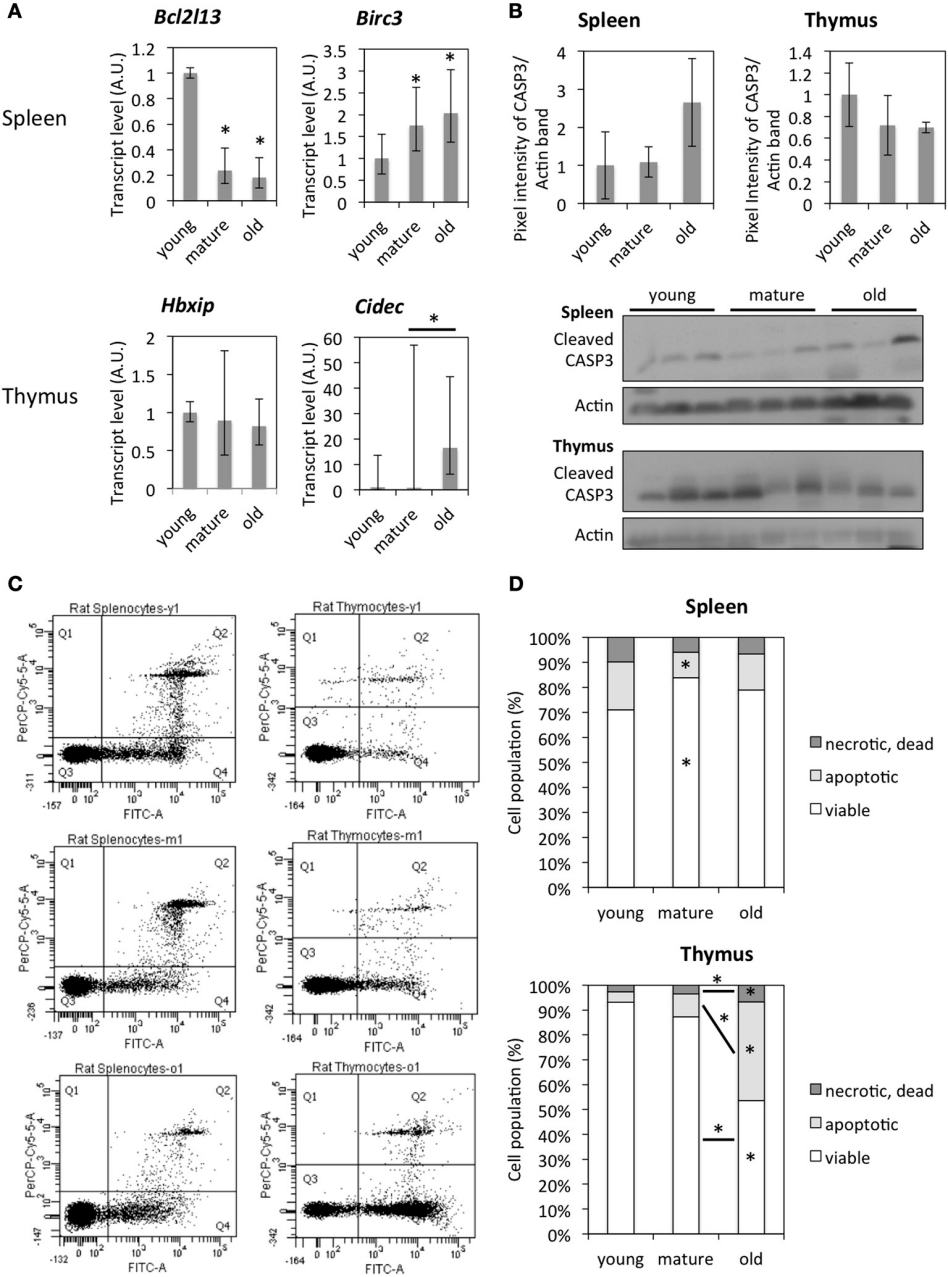
**Age-dependent changes in the activity of the apoptotic pathway. (A)** mRNA expression levels of genes involved in the apoptotic pathway by qRT-PCR. The data points represent means normalized to the expression in young animals ± 95% confidence intervals. **(B)** Western blot images and quantification of cleaved CASP3 band intensity normalized to Actin levels and protein levels detected in tissues from young animals. Each data point represents mean ± standard deviation. **(C)** Plots of cell populations were measured by flow cytometry, the PerCP-Cy5 channel detected PI-positive cells, and the FITC channel detected Annexin V-FITC positive cells. **(D)** Quantification of flow cytometer analysis of Annexin V/PI staining. The data points represent averages of 6–8 animals with 10,000 events detected per animal. The asterisks on bars indicate significant differences between young animals, and the asterisks above lines indicate significant differences between the two samples. ^*^Indicates significance based on one-way ANOVA (*p* < 0.05) in panel **(A,D)**.

First, expression levels of some pro-apoptotic genes (*Bcl2l13, Cidec*) and anti-apoptotic genes (*Birc3, Hbxip*) were confirmed by qRT-PCR (Figure 7A).

In order to study the effect of such gene expression changes on the function of encoded proteins, tissues were analyzed for their content of apoptotic cells by Western blot (cleaved Caspase 3) and Annexin V/PI staining followed by flow cytometer analysis. Western blot analysis for cleaved CASP3 revealed no significant differences among all three age groups either in the spleen or thymus, with a trend to an increased amount of cleaved CASP3 in the spleen of old animals and a decreased amount of that in the thymus (Figure 7B). Annexin V/PI staining of spleen cells indicated a significantly lower amount of apoptotic cells in mature compared to young tissues. Due to individual differences between animals, age-dependent differences were not significant (Figures 7C,D). However, the thymus of old animals contained a significantly higher fraction of apoptotic and dead cells compared to that of young and mature animals (Figures 7C,D).

### The composition of T cell populations in the thymus and spleen changes with age

The gene expression analysis indicated that there was an increased expression of *Cd4* in the spleen and a reduced expression of *Cd8a* and *Cd8b* in the thymus of older animals (S1). Changes in the expression of *Cd4, Cd8a*, and *Cd8b* indicated that T cell populations in both tissues would have altered composition in aging individuals.

**Figure 8:**
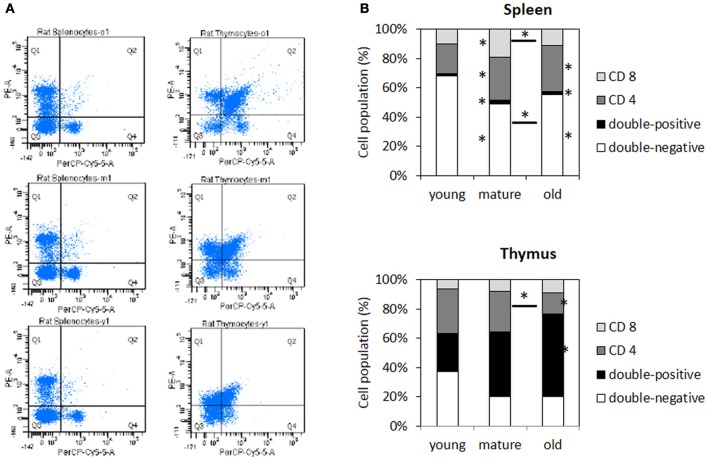
**Changes in the composition of T cell populations in the spleen and thymus of aging animals. (A)** Representative plots of PE-CD4 vs. PerCP-CD8A. **(B)** The bar graphs represent the percentage of the total cell populations in four different quadrants. Each data point represents an average of 6–8 samples with 10,000 events detected per animal. The asterisks next to the bars refer to significant differences to young animals, whereas the asterisks above lines indicate significant differences between mature and old animals. ^*^indicates significance based on one-way ANOVA (*p* < 0.05) in panel **(B)**.

Analyzing immune cell populations by a flow cytometer-based assay, in the spleen, we found smaller populations of CD4^−^CD8^−^ cells but slightly larger populations of CD4^+^CD8^+^ cells and CD4^+^CD8^−^ cells (Figure 8). In the thymus, individual differences were larger, but there was a significantly increased population of CD4^+^CD8^+^ cells in older animals along with a decrease in CD4^+^CD8^−^ populations (Figure 8). Furthermore, those changes were observed in the spleen of mature and old animals, but only in the thymus of old animals.

## Discussion

Here, we show that the timing of aging in immune organs is organ-specific and is accompanied by profound changes in epigenetic and transcriptional regulation of gene expression and thus gene expression profiles.

Increased expression levels of p16, along with decreased expression of CDK2, PCNA, and *Rrm2* in the spleen of mature and old animals and in the thymus of old animals (though mostly not significant in the thymus) indicates the accumulation of cells in G0/G1 phase of the cell cycle in those tissues. This, together with senescence-like gene expression patterns in the same tissues, suggests that senescent cells accumulate in the spleen of mature and old animals and in the thymus of old ones. Such age-dependent accumulation of senescent cells was previously observed in other species (Herbig et al., 2006) and has been associated with aging of the immune system (Aw et al., 2008).

Further, most cyclin genes were down-regulated in tissues of old animals (including *Ccna2, Ccnb2, Ccne1*, and *Ccne2*) except *Ccnd1*, which is a G1-cyclin and has also been shown to play a role in transcriptional regulation independent of CDKs (Coqueret, 2002). Therefore, the up-regulation of *Ccnd1* in old tissues may play a role in the inhibition of cell division (Casimiro et al., 2012), but this requires further study.

Thymus involution has previously been linked to the deregulation of several transcription factors—E2A, FOXN1 (Ortman et al., 2002) and NF-κB (Trebilcock and Ponnappan, 1996). Considering the high number of age-dependent gene expression changes observed, it is likely that more than one transcriptional program is affected. Therefore, bioinformatics analysis was performed to detect TFBS in the promoter regions of genes with changed expression; only those transcription factors that had the same directionality of expression changes as targeted genes were taken into consideration. Some limitations of this study were the limited availability of promoter sequences for genes represented on the expression BeadChip and of sequence information for TFBS. Nevertheless, this study identified several transcription factors correlating with the expression of their predicted targets. For some of those transcription factors, links with senescence, aging or lifespan variations have been made: BCL6 inhibits senescence (Shvarts et al., 2002); reduced MYC expression induces senescence (Guney et al., 2006); FOXO3 (Byun et al., 2013) and NF-Y are up-regulated with age (Matuoka and Chen, 2002). Among the transcription factors identified, BCL6, MYC, TCF7, and ETS1 may be interesting candidates for further studies in terms of immunosenescence because they exhibit opposing expression patterns in the thymus and spleen. Since decreased thymic output correlates with increased peripheral T cell proliferation (Mackall et al., 1993), the function of those transcription factors may give hints about the underlying mechanisms.

In addition to being controlled by transcription factors, expression profiles are modulated through epigenetic mechanisms. The gene expression analysis revealed the down-regulation of DNA methyltransferases in the spleen of mature and old rats and in the thymus of old rats, which was confirmed at the protein level and correlated with genome hypomethylation in the thymus but not spleen of mature and old animals. A decrease in genome methylation with age has been previously described in several species (Vanyushin et al., 1973; Wilson et al., 1987). Tissue-specific differences have been observed less frequently, but another study done on rats described non-significant DNA methylation changes in adipose tissues with age (Thompson et al., 2010).

Another heterochromatin mark—H3K9me3—has also been previously observed to be decreased in aging humans (Scaffidi and Misteli, 2006) but increased in flies (Wood et al., 2010) and mice (Braig et al., 2005). Braig et al. (2005) further determined H3K9me3 as a critical mark of senescence. However, in the spleen of mature and old rats and in the thymus of old rats, Suv39h1—a mediator of H3K9 methylation—was down-regulated at the RNA and protein levels and correlated with decreased levels of H3K9me3 in the spleen of mature and old rats and had a trend to low levels of H3K9me3 in the thymus of old rats. Therefore, in terms of H3K9 trimethylation, aging in rats is more similar to that in humans than in mice. The reduction in DNA methylation and H3K9me3 observed with increasing age suggests the loss of heterochromatin with age. However, no increase in euchromatin marks (histone acetylation) was detected. On the contrary, H4K16 acetylation levels were significantly reduced in the thymus of old compared to young animals. This is possibly due to the deregulation of both histone acetyltransferases (NuA4 complex) and histone deacetylases. Further, H4K16 is a target for deacetylation by *Sir2* in aging yeast (Dang et al., 2009), and therefore, acetylated H4K16 accumulates in aging yeast. However, we observed an opposite trend since mammalian genomes encode 7 sirtuin genes, and only *Sirt2* was down-regulated with age, which makes it possible that other histone deacetylases are responsible for the reduced levels of H4K16ac observed.

DNA hypomethylation and reduced levels of H3K9me3 may underlie some of the gene expression changes observed, but they might also affect silencing of repetitive sequences and miRNA expression. However, no significant induction of LINE1 ORF1 expression at either RNA or protein levels could be detected in the thymus and spleen of old rats. While several studies have found age-related changes in miRNA expression (reviewed in Grillari and Grillari-Voglauer, 2010), we did not study changes in miRNA expression profiles in aging rats. However, the results of gene expression analysis pointed toward changes in siRNA/miRNA processing and function with increasing age, with several genes involved in those processes being deregulated: *Adar, Ddx6, Fus, Piwi4l, Rbm3, Tardbp, Zcchc11*.

The expression of numerous DNA repair genes confirmed for *Tdg, Lig4*, and *Msh2* decreased with increasing age, suggesting a decreased efficiency/functionality of diverse repair pathways—BER, NER, MMR, HR, and NHEJ. This is supported by similar findings in various species and tissues [as reviewed by (Gorbunova et al., 2007)].

Both reduced expression of DNA repair genes and genome de-heterochromatinization, may predispose cells to genomic instability. This was observed in the thymus of mature and old animals in the form of longer Olive comet tails that indicate a higher amount of DNA strand breaks and a higher percentage of cells with γH2AX foci that indicate sites of unrepaired DNA damage. On the other hand, in spleen tissues of the same animals, opposite trends were observed which correlate with a slight increase in DNA methylation detected in those tissues.

Those molecular changes are also reflected in changing functional characteristics of spleen and thymus of older animals. An increase in the number of apoptotic and dead cells in the thymus was significant after 4 months of age. This is in line with previous reports that showed that age-related thymus involution occurs in rats between the ages of 5 and 12 months (Brelinska et al., 2008) as well as a study that demonstrated the accumulation of apoptotic cells in the epithelial compartment of the thymus (Aw et al., 2008). In the spleen, the individual differences were larger than age-related differences in the ratio of apoptotic and dead cells. Further, changes in the composition of T cell populations, which are considered to be a major feature of immunosenescence were observed in both tissues. In the thymus, a slight decrease in the double-negative T cell population and a significant decrease in CD8+ along with a prominent peak in the double-positive T cell populations were observed, while in the spleen, there was a significant decrease in the double-negative T cell populations along with an increase in the double-positive and CD4+ T cell populations. Interestingly, changes in the thymus occurred after maturity during thymic involution, while changes in the spleen occurred prior to maturity and thymic involution.

In addition to the genes and processes discussed here, a large variety of other genes were affected by expression changes, including many genes involved in cell signaling and immune function. Therefore, further study of how diverse signaling pathways are affected by expression changes may give insight into external regulation of those molecular events. Modeling of combined effects of the deregulation of several transcription factors may help in dissecting the contributions of intrinsic factors; and on an epigenetic level, ChIP studies of DNA regions enriched/depleted in DNA methylation or histone methylation marks in old vs. young tissues may elucidate the role of epigenetic regulation in immunosenescence.

**Figure 9:**
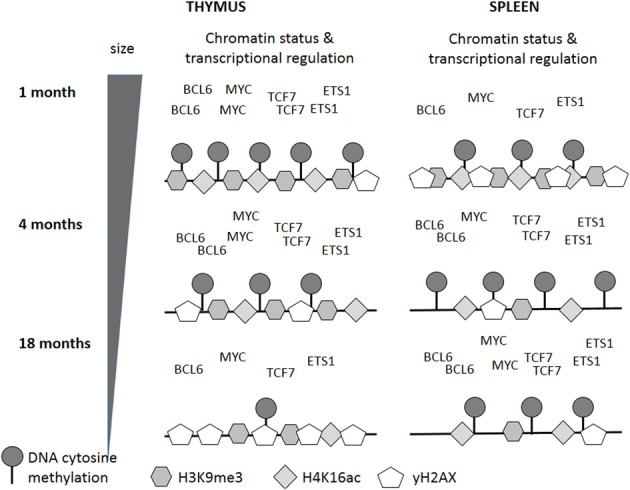
**Changes in epigenetic and transcriptional regulation may underlie age-related changes in genome stability and organ function**. The black lines indicate DNA, the numbers of symbols correlate with the experimental data and are relative to the presence of the corresponding marks in young animals. Transcription factor names occur relative to expression levels and are also relative to the expression in young animals.

In summary, our study shows that profound changes in mRNA expression profiles occur in the spleen and thymus of individuals at different chronological ages. Interestingly, these changes occur earlier in the spleen than in the thymus; and in the thymus, these changes seem to go along with thymic involution. This may be caused partially by altered expression of transcription factors and altered epigenetic profiles (Figure 9). Further, in the thymus, DNA and histone hypomethylation correlate with genomic instability and increased incidence of apoptosis and cell death, thus supporting the hypothesis that the molecular mechanisms underlying thymic involution may resemble those ones associated with the aging process in other organs.

## Methods

### Animal model and tissue sampling

Twenty male Long Evans rats, raised and bred at the local vivarium, were part of this study. Handling and care of animals were conducted in accordance with the recommendations of the Canadian Council for Animal Care and Use. The University of Lethbridge Animal Welfare Committee has approved all procedures. Animals were housed in a pathogen-free controlled facility at a 12 h light/dark cycle and given food and water *ad libitum*.

Animals were humanely sacrificed at the age of 1 month (6 animals), 4 months (6 animals), or 18 months (8 animals). Those time points were chosen as they represent different stages in the life of a rat: after weaning (1 month), early stage of thymic involution (4 months) and late stage of thymic involution (18 months).

Spleen and thymus cells were isolated as described for the comet and flow cytometer assays, and tissue samples were snap-frozen for RNA, protein, and DNA extraction.

### Gene expression profiling

RNA samples of the thymus of 2 animals and the spleen of 3 animals per group were prepared using Trizol Reagent (Invitrogen). Gene expression profiles were determined using Illumina® RatRef12 Gene Expression BeadChips. Differential expression analyses were performed by the Illumina® GenomeStudio software using an Illumina-custom model with an FDR applied. Only genes for which the differential expression analysis was significant based on *p* < 0.05 and a log2-fold change cut-off of −0.4/0.4 were considered for further analysis.

#### Functional classification of gene expression data

The bioinformatics analysis performed included Sample Clustering, which was performed using the Illumina® GenomeStudio Software, and functional classification of genes. The functional classification of genes was done using FunNet Transcriptional Networks Analysis (www.funnet.info) and DAVID Bioninformatics Resources 6.7 (Huang da et al., 2009).

#### Analysis of promoters for transcription factor binding sites (TFBS)

Promoter regions of 1000 bases upstream of the transcription start site of differentially expressed RefSeq genes were extracted from the March 2012 (RGSC 5.0/rn5) rat genome assembly (Gibbs et al., 2004). TFBS were retrieved from Gencards (http://www.genecards.org) (Safran et al., 2010) or Bio-Base (https://portal.biobase-international.com/). The promoter sequences were then searched for TFBS using the PatMatch v1.2 program (Yan et al., 2005), with allowing no mismatches.

The number of occurrences of a TFBS in the promoters of differentially expressed genes was compared to its occurrences in the promoters of all genes in the rat genome assembly. Only genes, for which the number of TFBS occurrences within their promoter was higher or lower than mean ± standard deviation number of occurrences in promoters of all the genes in the rat genome were considered for further analysis. Transcription factor—target gene correlation was performed to check whether expression changes in transcription factors coincided with expression changes in target genes. Target genes, for which such a correlation was found, were considered for further analysis.

### qRT-PCR

The validation of gene expression results was done by qRT-PCR. qRT-PCR reactions were set up using the SsoFast™ EvaGreen® Supermix (BioRad) and analyzed according to SSoFast guidelines with annealing temperatures as specified for specific primer pairs (Table 4).

**Table 4:** **Primers used for qRT-PCR analysis of transcript levels**.

**Gene**	**Fwd/rev**	**Sequence**	**Annealing temperature**
*Suv39h1*	Fwd	5′-ATGCTGGCTAATACTAAC-3′	56.9°C
	Rev	5′-TCTACTTCCTGATGGTAA-3′	
*Lig4*	Fwd	5′-ATACAGAAGGTGAATGAA-3′	56.9°C
	Rev	5′-TGGAAGATGGACAATATC-3′	
*Cidec*	Fwd	5′-CTGAACTGAATGGACATAG-3′	56.9°C
	Rev	5′-TTACCGCAGACTCTAATG-3′	
*Hbxip*	Fwd	5′-GCCATTCACTTAATGTCCAAT-3′	59.6°C
	Rev	5′-CCACCACACTCCTAACTG-3′	
*Bcl2l13*	Fwd	5′-TCTACGACTGTGCCTCTA-3′	59.6°C
	Rev	5′-GGGACCTTGTTGTTTCTC-3′	
*Birc3*	Fwd	5′-TCAGAGCACAGGAGACAT-3′	56.9°C
	Rev	5′-TCAGGTTAGAGACAGTGTATTG-3′	
*Tdg*	Fwd	5′-TTCTCTGACTTGATGGTA-3′	56.9°C
	Rev	5′-TCATAGAACAGGCTACAT-3′	
*Rrm2*	Fwd	5′-TTAGCCAAGAAGTTCAAG-3′	56.9°C
	Rev	5′-AATGTAAGTGTCAATAAGGA-3′	
*Cdc25b*	Fwd	5′-GTGAAGAAGATGACGGATT-3′	56.9°C
	Rev	5′-TGGAGCACTAATGAGGTT-3′	
*Cd4*	Fwd	5′-AAGGTGAAGAGGTCAAGATG-3′	56.9°C
	Rev	5′-CAGCCAGGAACATTGTCT-3′	
*Cd8a*	Fwd	5′-TATTGTCCTCTGTATTGTT-3′	56.9°C
	Rev	5′-GACTATCTCTGGTGTTAC-3′	
*Ctgf*	Fwd	5′-TGTGCCTATTGTTCTTGT-3′	59.6°C
	Rev	5′-CAGTCACTCAGGTTACAG-3′	
*Pot1a*	Fwd	5′-TAAGCCTCCATATCTCAG-3′	56.9°C
	Rev	5′-GAACAATGTCTCCAACTT-3′	
*B2m*	Fwd	5′-TTAGCAGCCTAGCAGTTC-3′	61.2°C
	Rev	5′-ACCACTTCACTTCACTCTG-3′	
*Hprt*	Fwd	5′-TGACTATAATGAGCACTTC-3′	56.9°C
	Rev	5′-AGGACTCTTGTAGATTCA-3′	
*Hspcb*	Fwd	5′-TGAGAACAAGAAGAAGAAGAA-3′	56.9°C
	Rev	5′-ACGGATGAAGTTGAGGTA-3′	
*RplP1*	Fwd	5′-GGTCACGGAGGATAAGAT-3′	56.9°C
	Rev	5′-ATGAGGCTTCCAATGTTG-3′	
*Ldha*	Fwd	5′-CGAGAGCATAATGAAGAAC-3′	53.5°C
	Rev	5′-TCCTTGATTCCATAGAGAC-3′	

Each experiment included three biological replicates with 2 technical replicates each. *B2m, Hprt, Ldha, Hspcb*, and *RplP1* were used for the normalization, which was carried out using qbase^PLUS^ (Vandesompele et al., 2002).

### Western immunoblotting

Protein preparations were obtained by sonicating approximately 50 mg of spleen or thymus tissues in 100 μL of cold 1% SDS containing protease inhibitor (Roche). Western immunoblotting was performed as previously described (Pogribny et al., 2005) using the primary antibodies specified in Table 5. Chemiluminescence was detected using a FluorChem HD2 camera with FluorChem software (Cell Biosciences). To confirm equal loading, membranes were stained with Coomassie blue (BioRad). Signals were quantified using the NIH Image J64 software and normalized relative to Actin or Coomassie staining as indicated.

**Table 5:** **Antibodies used for Western blots**.

**Target**	**Supplier, cat no**	**Dilution**
Mouse anti-CDK2	Santa Cruz, sc6248	1:500 in 5% milk (PBST)
Mouse anti-p16	Santa Cruz, sc1661	1:500 in 5% milk (PBST)
Mouse anti-PCNA	Santa Cruz, sc56	1:1000 in 5% milk (PBST)
Mouse anti-DNMT1	Abcam, ab13537	1:500 in 5% milk (PBST)
Rabbit anti-DNMT3A	Santa Cruz, sc20703	1:500 in 5% milk (PBST)
Rabbit anti-SUV39H1	Abcam, ab33056	1:500 in 5% milk (PBST)
Rabbit anti-SIRT2	Santa Cruz, sc20966	1:500 in 5% milk (PBST)
Rabbit anti-H3K9me3	Abcam, ab8898	1:500 in 5% milk (PBST)
Rabbit anti-H3	Cell Signaling, 9715	1:1000 in 5% milk (PBST)
Rabbit anti-H4K16ac	Cell Signaling, 8804	1:1000 in 5% milk (PBST)
Mouse anti-H4	Cell Signaling, 2935	1:1000 in 5% milk (PBST)
Rabbit anti-MSH2	Santa Cruz, sc494	1:500 in 5% milk (PBST)
Mouse anti-RAD51	Santa Cruz, sc56212	1:500 in 5% milk (PBST)
Rabbit anti-Cleaved CASP3	Cell Signaling, 9664	1:500 in 5% milk (PBST)
Mouse anti-ACTIN	Abcam, ab3280	1:1000 in 5% BSA (PBST)
**SECONDARY ANTIBODIES**		
Donkey anti-Rabbit	Santa Cruz, sc2313	1:10000 in 5% milk (PBST)
Goat anti-Mouse	Santa Cruz, sc2005	1:5000 in 5% milk (PBST)

### Cytosine extension assay

DNA was isolated from approximately 25 mg of spleen or thymus tissues using the DNeasy® Blood and Tissue Kit (Qiagen). A Cytosine Extension Assay was performed as previously described (Boyko and Kovalchuk, 2010). CPM values were determined using a PerkinElmer Liquid Scintillation Analyzer Tri-Carb 2910 TR.

### DNA damage and repair assays

#### Comet assay

The isolation of cells from spleen and thymus tissues for comet assays was performed in mincing buffer (110 mM NaCl, 4 mM KCl, 0.35 mM KH_2_PO_4_, 0.27 mM Na_2_HPO_4_, 0.08% Glucose, 3.33 mM NaHCO_3_, 25.5 mM EDTA, 10% DMSO, pH 7-7.5) by grinding tissue pieces through 40 μm Nylon meshes. Comet assays were performed as described previously (Olive and Banath, 2006). For scoring comets, the slides were stained with 1:1000 Sybr Gold and viewed using a Zeiss Observer Z1 microscope and a Stingray CCD camera (Allied Vision Technologies). The comets were evaluated using the Comet Assay IV software (Perceptive Instruments Ltd.). The values of the Olive Tail moment were used as a measure of DNA damage. The experiment included three independent biological replicas, each including two technical repeats. In total, 300–400 comets were analyzed per experimental group.

#### γH2AX/PCNA immunofluorescence staining

Touch prints of spleen and thymus tissues were performed on positively charged microscope slides (VWR) and fixed in 4% PFA for 20 min. This was followed by washing in PBS for 20 min and storing in 70% Ethanol.

Immediately before staining, the touch prints were washed in PBS and permeabilized in ice-cold Methanol. This was followed by blocking 10% BSA/10% Goat Serum/0.1% Tween20 in PBS for 2 h and incubation with primary antibody (rabbit anti-γH2AX (1:100, Cell Signaling) and mouse anti-PCNA (1:200, Santa Cruz) in blocking solution) overnight at 4°C. Antibody binding was detected by incubation with secondary antibodies AlexaFluor® 488 goat anti-rabbit and AlexaFluor® goat anti-mouse (1:700, Invitrogen) in blocking solution. The slides were counterstained with Hoechst 33342. Following dehydration in increasing Ethanol concentration, he slides were air-dried and mounted in 50% Krystalon/50% Ethanol and viewed using a Zeiss Observer Z1 epifluorescence microscope with AxioVision Rel 4.8 software.

### Flow cytometer assays

#### Cell isolation

Cells from spleen and thymus were obtained by grinding tissue pieces through a 40 μm Nylon mesh in HyClone® DMEM High Glucose (Fisher Scientific) containing 10% FBS (Gibco). The cells were washed and resuspended in 1 ml PBS in the case of cells isolated from thymus or in the case of spleen cells incubated in 500 μ l ACK Lysis Buffer (0.15 M NH_4_Cl, 1 mM KHCO_3_, 0.1 mM Na_2_EDTA, pH 7.2) for 10 min. Then the cells were supplemented with 9 ml of medium, pelleted and resuspended in 1 ml PBS.

#### Detection of apoptotic, necrotic, and dead cells

In order to determine the ratios of apoptotic, necrotic and dead cells in thymus and spleen tissues, cells were stained using the FITC Annexin V Apoptosis Detection Kit (BD Pharmigen™) according to the manufacturer's instructions and analyzed using the BD FACS Canto II (BD Biosciences). Ten thousand events were detected for each sample.

#### Detection of CD4 and CD8 cell surface markers

For the detection of CD4 and CD8 antigens, the cells were washed twice with IFWB (4% FBS/0.02% NaN_3_ in PBS) followed incubation with primary antibodies PE mouse anti-rat CD4 and PerCP mouse anti-rat CD8a (0.09 μg each, BD Pharmigen) for 45 min. Cells were then washed twice in IFWB and fixed in 100 μl 4% PFA in PBS for 1 h at 4°C. The cells were washed in 500 μl of IFWB, resuspended in 500 μl PBS and analyzed on the BD FACS Canto II (BD Biosciences). Ten thousand events were detected for each sample.

### Statistical treatment of data

Results are presented as mean of at least three animals per group with standard deviation or 95% confidence interval as indicated. A statistical analysis of results was performed using One-Way ANOVA (*p* < 0.05) in the case of qRT-PCR experiments or two-tailed Student's *t*-test (*p* < 0.05) for the analysis of all other presented results as indicated.

### Conflict of interest statement

The authors declare that the research was conducted in the absence of any commercial or financial relationships that could be construed as a potential conflict of interest.

## Supplementary material

Figure S1 **Gene expression data (link to website to be provided)**.

Figure S2 **Gene Ontology terms overrepresented in the Gene Expression Data Set**.

Figure S3 **Age-dependent changes in SIRT2 protein levels and associated histone acetylation**.
