# Supplementary figures and images for: Immunosenescence is associated with altered gene expression and epigenetic regulation in primary and secondary immune organs
Source: Front Genet. 2013 Oct 18;4:211. doi: 10.3389/fgene.2013.00211 (PMC3798808; doi:10.3389/fgene.2013.00211)

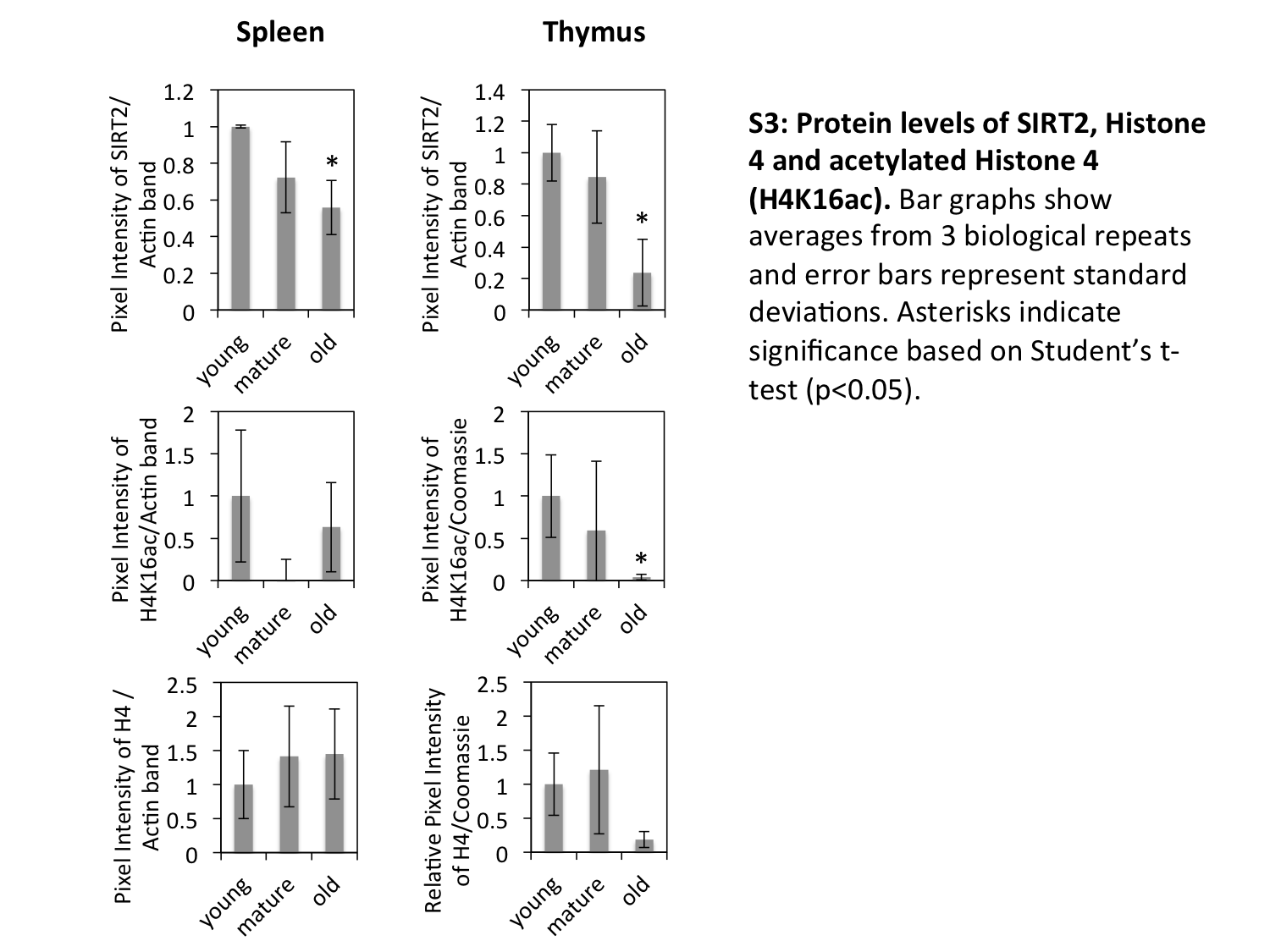

Supplement: Figure S1 — Gene expression data (link to website to be provided). [file Presentation1.ZIP › 63079_Kovalchuk_S3.TIFF]
